# Habitat-use of the vulnerable Atlantic Nurse Shark: a review

**DOI:** 10.7717/peerj.15540

**Published:** 2023-06-15

**Authors:** Vanessa Brito Bettcher, Ana Clara Sampaio Franco, Luciano Neves dos Santos

**Affiliations:** 1 Theoretical and Applied Ichthyology Lab (LICTA), Federal University of the State of Rio de Janeiro, Rio de Janeiro, RJ, Brazil; 2 Postgraduate Program in Ecology and Evolution (PPGEE), Rio de Janeiro State University (UERJ), Rio de Janeiro, RJ, Brazil; 3 Postgraduate Program in Neotropical Biodiversity (PPGBIO), Universidade Federal do Estado do Rio de Janeiro (UNIRIO), Rio de Janeiro, RJ, Brazil

**Keywords:** Conservation, Ecosystem, Habitat association, Marine protected areas

## Abstract

Human activities have led to the loss of critical habitats for aquatic species at such an accelerated rate that habitat modification is considered a leading threat to biodiversity. Sharks and rays are considered the second most threatened group of vertebrates that have also suffered from habitat loss, especially in nursery grounds and reef-associated species. In this sense, actions toward the conservation of critical grounds for species survival are urgently needed, especially for those threatened with extinction. This study aimed to gather and provide information on the worldwide distribution and habitat association of the *‘vulnerable’* Atlantic Nurse Shark *Ginglymostoma cirratum* through a literature review performed at the Dimensions research database. A total of 30 studies published between 1950 and 2021 were retained since they defined at least the type of habitat in which *G. cirratum* was associated. Most studies covered the Floridian ecoregion, where *G. cirratum* is more common and abundant. Reefs, seagrass, sandy, rocky, mangrove, and macroalgae accounted for the majority of habitat associations, with a higher diversity of habitats detected within marine protected areas (MPAs). *Ginglymostoma cirratum* was recorded at a maximum depth of 75 m, temperatures ranging from 25 °C to 34 °C, and salinities between 31 and 38 ppt. Neonates were associated with shallower habitats (<20 m), mostly reefs, rocks, macroalgae, sandy shores, and seagrass, in an average temperature of 26 °C and salinity of 36 ppt. Breeding events and habitats were reported by 11 studies, 72.7% of them in shallow waters, mostly inside MPAs (90.9%). Our findings highlighted the key role played by MPAs in protecting essential grounds for threatened species, such as the Atlantic Nurse Shark. Major ecoregions (*e.g*., the Eastern Atlantic) are still underrepresented in the scientific literature as long as studies aim specifically to assess *G. cirratum* habitat association. Thus, further insights into the essential habitats needed to conserve the Atlantic Nurse Shark can still emerge from future studies. Considering the recent IUCN extinction risk status change in *G. cirratum* (*i.e*., *Data Deficient* to *‘Vulnerable’*), new conservation measures that integrate habitat protection and management are urgently needed and should consider the data collected herein.

## Introduction

Species occurrence is an expression of the “fundamental niche” which reflects the physiological limits imposed for a given species by climatic and chemical parameters (Soberón & Peterson, 2005). Within favorable conditions, species select essential habitats based on their evolutionary history, but also in pursuit of prey abundance, predator risk, and refuge availability (Soberón & Peterson, 2005). The assessment of species-habitat associations enables modeling species’ potential distribution, especially for endangered species, investigating possible causes of decline, or even predicting the effects of future habitat alterations on species distributions, supporting the creation of conservation plans that are more accurate under a future of global changes (Redhead et al., 2016). Thus, defining the physical conditions that enable species occurrence is essential to understanding the nature of those associations. Unlike in terrestrial systems, knowledge of marine habitat alteration effects is moving forward very slowly and remains barely known for most species, despite these systems being constantly subjected to several human impacts (*e.g*., fisheries, pollution, and acidification) (Airoldi, Balata & Beck, 2008).

Habitat is also a mediating factor underlying species co-occurrence, which reflects in increased reef fish abundance and richness in the presence of more rich and complex habitats (Chittaro, 2004). However, many human activities (*e.g*., urbanization, fisheries, and pollution) have been interfering with the quality, diversity, availability, and complexity of habitats in aquatic ecosystems. Consequently, these human activities also interfere with the composition and structure of fish assemblages, by potentially disrupting competitive and mutualistic fish relationships. Many of these complex interactions are the same which support ecosystem services, such as fisheries, erosion control, flooding, food production, and tourism, among others (Dulvy, Sadovy & Reynolds, 2003; Worm et al., 2006; Capitani et al., 2021). Elasmobranchs, mainly medium and large-size species, are commonly known for their role in structuring and connecting food chains and ecosystems such as through nutrient cycling, habitat modification, and biocontrol of weak and sick individuals, and invasive species by reef-associated mesopredators (Estes et al., 2016; Roff et al., 2016; Jorgensen et al., 2022). However, sharks and rays are among the most threatened vertebrates today, with 387 species listed as threatened in the IUCN Red List (Bascompte, Melian & Sala, 2005; Desbiens et al., 2021; Dulvy et al., 2021). In addition to fishing, which currently threatens 100% of those species, the group is threatened by habitat loss (31.2% of the threatened chondrichthyans), climate change, and pollution (Dulvy et al., 2021). Threats to elasmobranch habitat are represented by several categories in the IUCN classification. Still, they have potential synergistic effects, such as climate change that not only influence species’ thermal affinity and, consequently, their distribution but also threaten habitat conservation, leading to habitat degradation through coral bleaching (Dulvy et al., 2021).

Recently listed as *‘vulnerable’* (VU) on the IUCN Red List, the Atlantic Nurse Shark, *Ginglymostoma cirratum*, was included in this category due to threats mainly imposed by fishing and continuous habitat decline, both in extension and quality (Carlson et al., 2021; Ward-Paige et al., 2010). Therefore, the protection of essential areas is one of the conservation actions indicated for the protection of this species (Carlson et al., 2021), which frequently is achieved through the implementation of protected areas and no-take zones. Marine protected areas (MPAs) are often used as a strategy for elasmobranch conservation, especially to reduce the impact of fisheries, but also to preserve essential ecosystems for species at their different life stages (MacKeracher, Diedrich & Simpfendorfer, 2019). The urgency to identify and protect essential habitats for the species has been evidenced. The Shark Special Protection Zone in the Dry Tortugas’ courtship and the mating ground is one example of an MPA proposed by Carrier & Pratt (1998) that is closed for marine boat traffic during the breeding season of the Atlantic Nurse Shark. The success is evidenced by the mating site fidelity of some individuals (Pratt et al., 2022). Other evidence of MPAs’ importance to protect the Atlantic Nurse Shark is the recent discovery of their occurrence in its southernmost distribution, in a mosaic of no-take and sustainable use MPAs of São Paulo, Brazil, after being considered locally extinct (Rosa et al., 2006; Santos et al., 2023). Even though they have been proven to be essential for species protection, these areas are often fewer and smaller than necessary to cover the home range of certain species, including the Atlantic Nurse Shark (Ward-Paige, 2017; Dwyer et al., 2020), and are subjected to several political limitations, which may undermine their potential to protect threatened species. Dwyer et al. (2020) showed that 50 km long MPAs would be necessary to protect 50% of all Atlantic Nurse Shark local individuals from fishing. This is because contrary to what has been believed that this is a sedentary species, the Atlantic Nurse Shark showed mean weekly dispersal distances of 8.02 ± 5.03 km (Dwyer et al., 2020). Other records have also evidenced long-distance migrations of the species (541 km, Kohler & Turner, 2001; 335 km, Pratt et al., 2018) highlighting the need for a regional perspective on the protection of the Atlantic Nurse Shark.

The Atlantic Nurse Shark occurs from North Carolina (USA) to southern Brazil, colonizing several coastal ecosystems, such as sandy flats, coral reefs, seagrasses, and mangroves. However, the specific role played by each habitat during the life cycle of the species (*i.e*., mating, feeding, and recruitment) is essential to understand the distribution patterns and advising conservation agencies towards protection measures for this species. This is particularly crucial since the same habitats that are essential to *G. cirratum* are those that have been threatened by urbanization and ocean warming (Carlson et al., 2021; Capitani et al., 2021; Garzon et al., 2021), posing an even greater threat to this *‘vulnerable’* species. Considering (a) the challenge to obtain primary data on habitat use of such a largely distributed species, (b) habitat modification as one of the major threats to biodiversity and ecosystem functioning, (c) the conservation actions indicated for *G. cirratum*, and (d) the current demand for scientific data on ecosystem assessment, a literature review was carried out to provide information on ecological factors associated with the occurrence of *G. cirratum* populations and to point out and discuss priority areas for species conservation.

## Survey methodology

A literature review was performed at the Dimensions research database to assess the geographical distribution and essential habitats associated with the Atlantic Nurse Shark *G. cirratum*. The Dimensions research database is a recent alternative to perform reviews of the scientific literature more comprehensive than Web of Science and Scopus and less general than Google Scholar (Hook, Porter & Herzog, 2018; Martín-Martín et al., 2021; Singh et al., 2021). We used secondary data to address two questions: (1) the geographical distribution of the Atlantic Nurse Sharks; and (2) the type of habitats associated with the populations of the Atlantic Nurse Sharks. To achieve the first goal, a literature review was performed on 26th January 2021 using as keywords the scientific name of the species and its synonyms according to Froese & Pauly (2021) (see Appendix S1 for further details on keywords). We retained only articles that provided the exact location where the Atlantic Nurse Sharks were sampled or observed. To assess the association of the *G. cirratum* with habitats we performed another search on the 10th of August 2021 (see Appendix S2 for further details on keywords). We retained all the studies that described the habitat with which the Atlantic Nurse Shark was associated. The studies listed in the supporting information (SI) of the review performed by Osgood & Baum (2015) were also screened. Studies that did not describe the habitat of the study site where the sharks were collected/observed and those that we could not access were excluded from our database.

The habitat types were divided into six categories: Macroalgae (algae, benthic algae, seaweed, brown algae, and green algae); Mangrove (mangrove lined barrier islands, mangrove forests, mangrove-fringed bay, mangrove barrier, and mangrove islands); Reefs (reefs, flat reef, reef canyon, reef front, ocean reefs, fringing reefs, corals, coral bottom, corals and sponges, amidst colonies of octocorals, hard bottom, and calcareous algae); Rocky (rocky substrate, rocky shores, under rocks, boulders, cliffs, and channels); Sandy (oolitic sand banks, sandy shelf, sandy shoal, sand bottom, sandy substratum, and lagoon); and Seagrass (mud and patchy seagrass, and seagrass beds). We also recorded, from each retained study, the following information: study site, geographic coordinates, and whenever available, the sex ratio and total length of Atlantic Nurse Sharks, and water temperature, depth, and salinity. Sharks smaller than 31.5 cm TL were considered neonates, while individuals larger than 215.0 cm TL were classified as adults (Castro, 2000), regardless of sex (considering that most of the studies did not provide individual sizes per sex).

Publications were classified into their field of research according to the Australian and New Zealand Standard Research Classification (ANZSRC) used by Dimensions. We used Google Earth to estimate the geographical coordinates of the occurrences when the authors provided only the name of the sampling site. The occurrences of the Atlantic Nurse Shark were assigned to ecoregions and provinces following the Marine Ecoregions of the World (MEOW) as proposed by Spalding et al. (2007) using the shapefile in Google Earth. Maps with the geographical distribution of *G. cirratum* were built using a shapefile of the MEOWs (downloaded from: https://www.worldwildlife.org/publications/marine-ecoregions-of-the-world-a-bioregionalization-of-coastal-and-shelf-areas) in Quantum GIS 3.16.1 (QGIS, 2022).

We also evaluated whether the study sites coincided with the limits of MPAs by overlaying a layer with the geographic coordinates of all study sites with the World Database on Protected Areas (UNEP-WCMC & IUCN, 2021). The Bahamian Shark Sanctuary was also considered a MPA. This evaluation was not performed in large-scale studies that did not provide the exact coordinate of the capture/observation, since we could not evaluate whether the sharks were sampled in or out of the MPAs limits. All habitat types and locations in the database were considered, whenever the article provided information on single or multiple locations with more than one habitat type.

## Results

**Figure 1:**
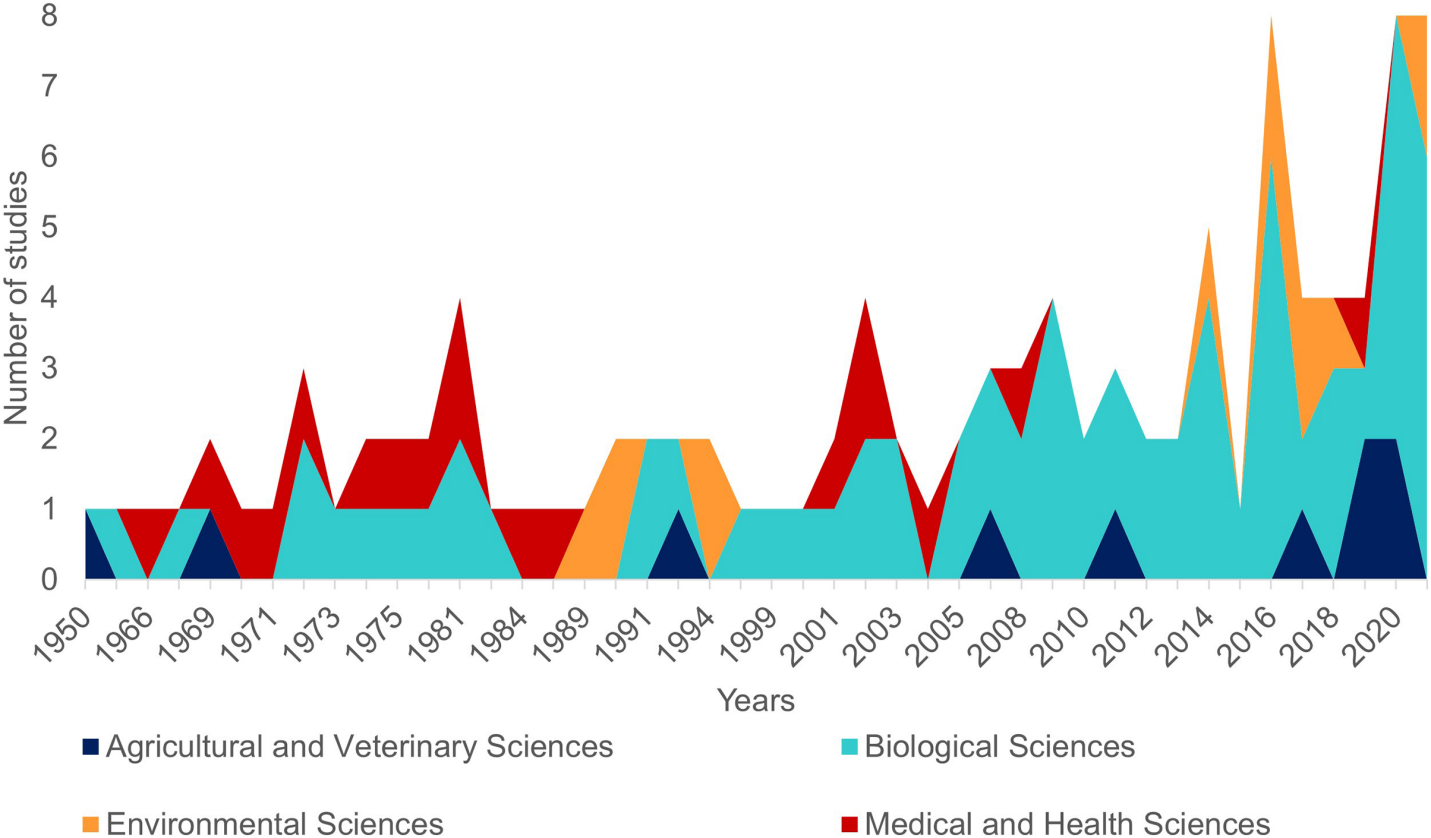
Number of publications per year dealing with the Atlantic Nurse Shark *Ginglymostoma cirratum* according to our review (see Methods for details). Colors indicate the field of research according to the Australian and New Zealand standard research classification (ANZSRC).

**Figure 2:**
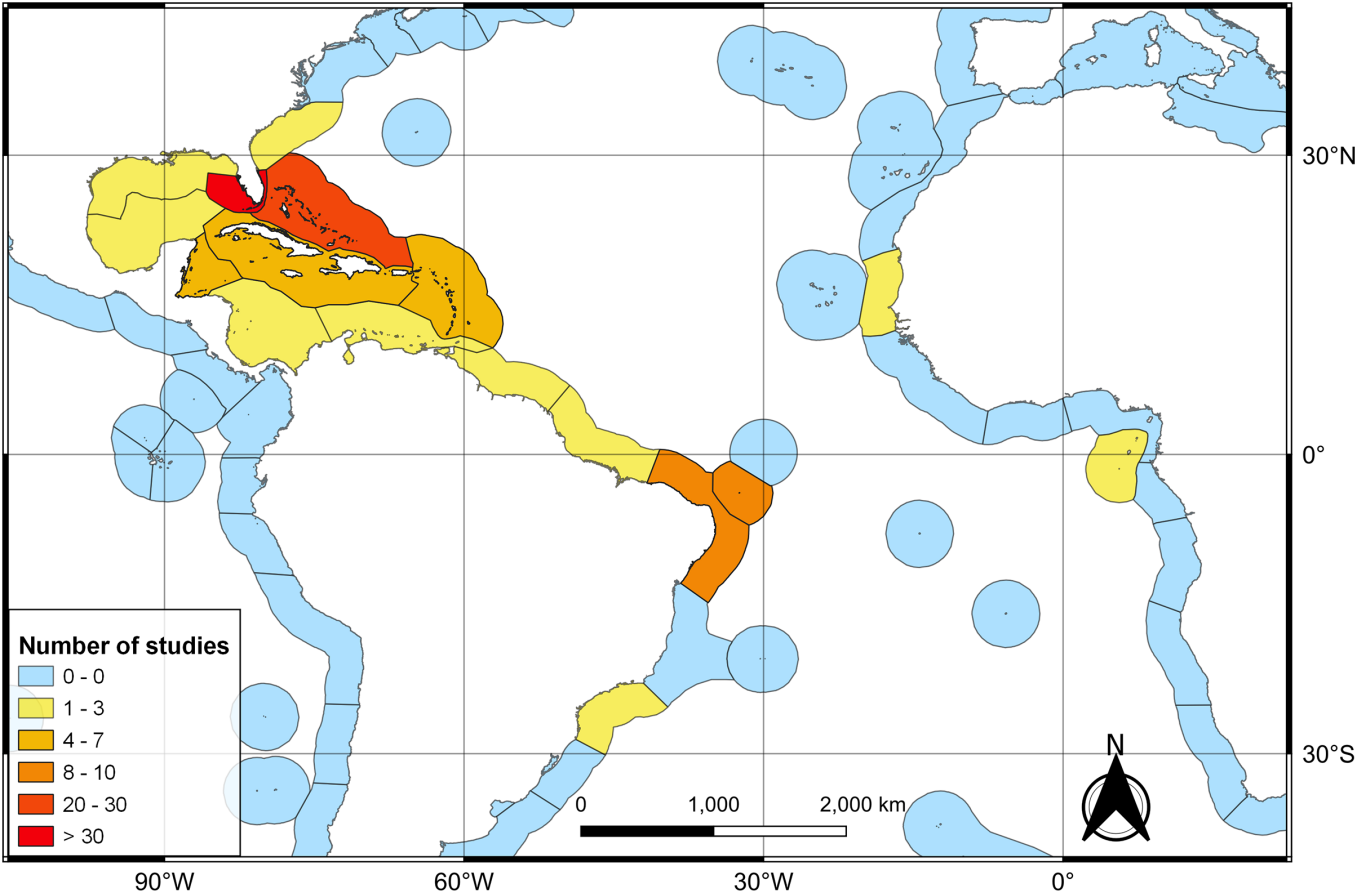
Heat map of the number of publications dealing with the Atlantic Nurse Shark *Ginglymostoma cirratum* per marine ecoregions of the world (MEOW).

The first literature review yielded 194 articles and 109 were retained to depict the geographic distribution of the Atlantic Nurse Shark (Appendix S1). Publications from the Southwestern Atlantic dated from 1967 to 2021 (*N* = 16) and from the Northwestern Atlantic from 1964 to 2021 (*N* = 93) (Fig. 1). Studies in the field of biological sciences accounted for most publications (62.4%), peaking in recent years. Most studies were performed in the Northwestern Atlantic (87.3%), particularly in the Floridian ecoregion (39.2%). Only two studies covered the Eastern Atlantic distribution of the Atlantic Nurse Shark (Trape, 2008; Karl, Castro & Garla, 2012) (Fig. 2).

**Figure 3:**
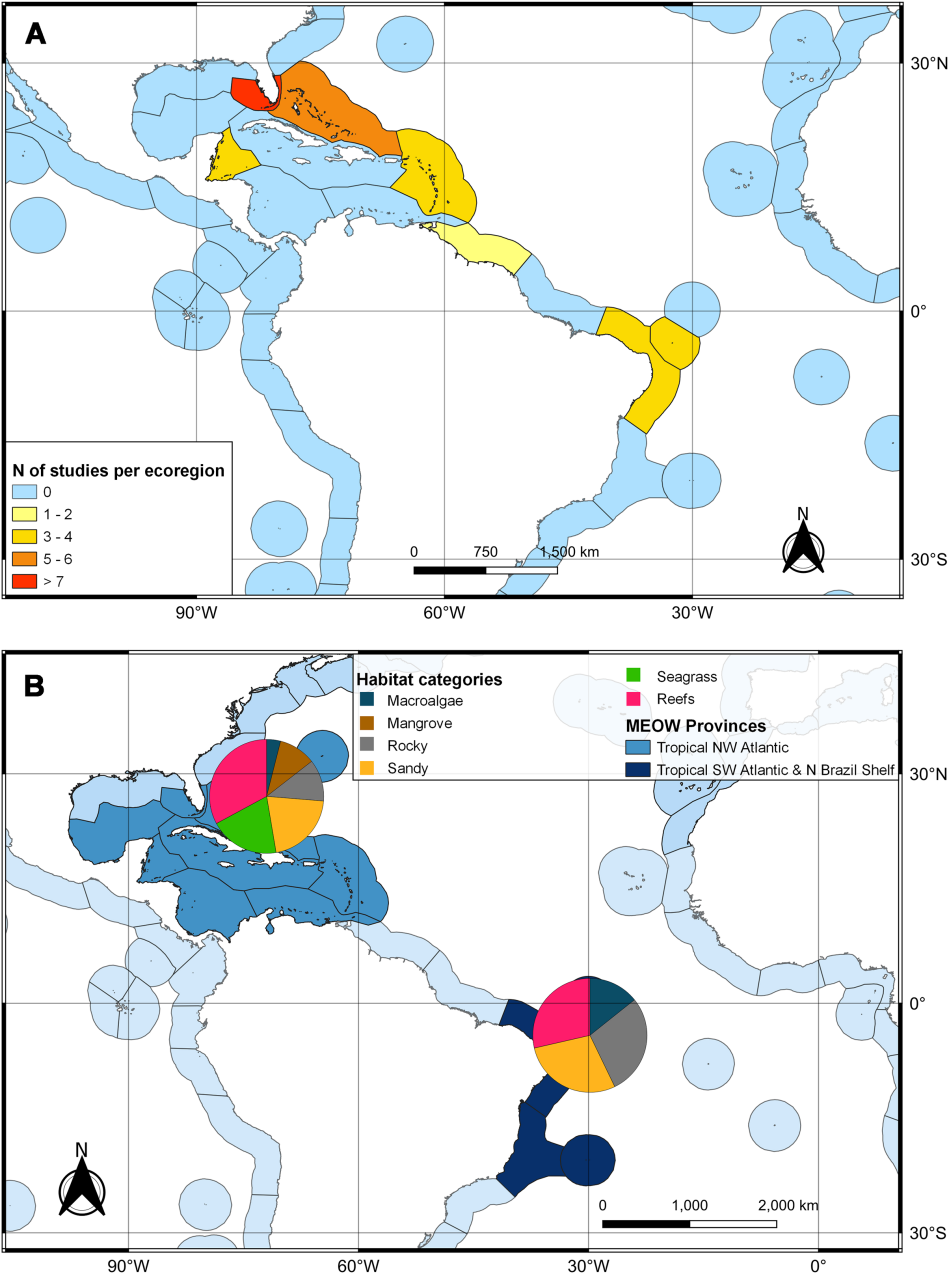
Heat map of the marine ecoregions of the world (MEOW) showing: (A) number of publications with Atlantic Nurse Shark habitat by ecoregion; (B) frequency of habitat types per Northern and Southern Atlantic provinces.

**Figure 4:**
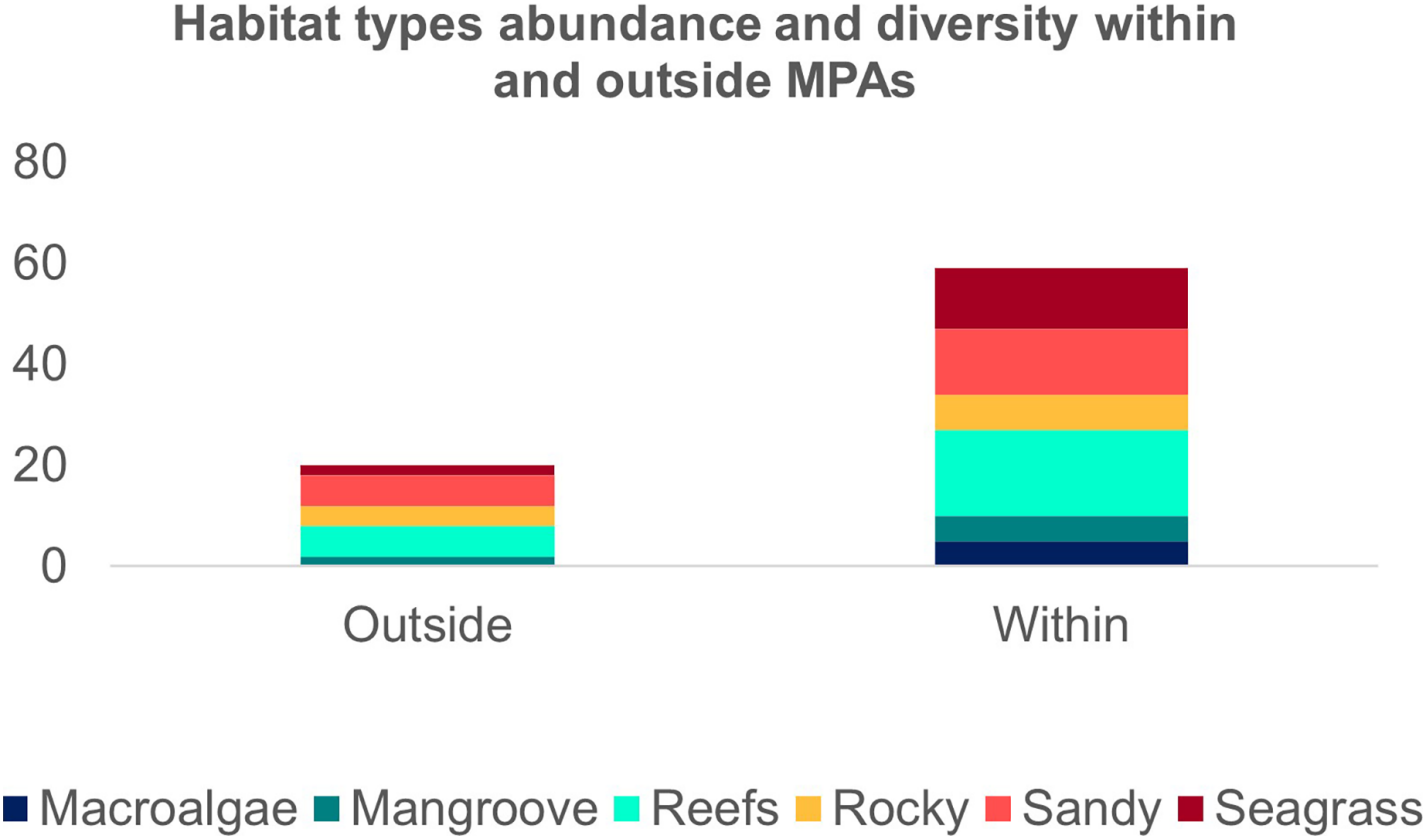
Frequency of the Atlantic Nurse Shark *Ginglymostoma cirratum* habitats within and outside marine protected areas.

The second search yielded 31 studies (29 articles and two book chapters) but excluded one article that referred to *Ginglymostoma unami* (Pacific nurse shark) and eight to *Carcharias taurus* (grey nurse shark). Six additional studies were included in our database due to previous knowledge of the authors that they dealt with Atlantic Nurse Shark habitats. Out of the 13 articles in the SI from Osgood & Baum (2015), three were already present in our database. We were unable to find five articles, and out of those, five did not address the Atlantic Nurse shark habitat. One article was excluded because it relied on secondary data, and as a result, we added Gilmore et al. (1977) to the database. The final database was composed of 30 articles. Studies on the habitat colonized by Atlantic Nurse Sharks were published between 1950 and 2021 (Appendix S2) and followed a similar pattern to the distribution studies. Most data were obtained from the Floridian ecoregion (*N* = 13; Gilmore et al., 1977; Smith & Herrkind, 1992; Carrier & Pratt, 1998; Castro, 2000; Wiley & Simpfendorfer, 2007; Pratt & Carrier, 2007; Adams & Paperno, 2007; Ward-Paige et al., 2010; Hannan et al., 2012; Pratt et al., 2018; Rangel, Hammerschlag & Moreira, 2021; Gutowsky et al., 2021; Tinari & Hammerschlag, 2021), followed by surrounding ecoregions, the Bahamian (*N* = 5; Hansell et al., 2018; Shipley et al., 2018; Ward-Paige et al., 2010; Bruns & Henderson, 2020; Lennon & Sealey, 2021), and Western Caribbean (*N* = 4; Eggleston & Lipcius, 1992; Chapman et al., 2005; Pikitch et al., 2005; Ward-Paige et al., 2010). There was also data from Northeastern Brazil (*N* = 3; Ferreira et al., 2013; Afonso, Andrade & Hazin, 2014; Niella, Hazin & Afonso, 2017), Fernando de Noronha and Atoll das Rocas (*N* = 3; Garla et al., 2009; Garla, Garrone-Neto & Gadig, 2014; Afonso et al., 2016), Eastern Caribbean (*N* = 3; Smith-Vaniz, Jelks & Rocha, 2006; DeAngelis et al., 2008; Stoffers et al., 2021), and Guianan (*N* = 1; Springer, 1950) ecoregions (Fig. 3A). A greater variety of habitats was detected at the Tropical Northwestern Atlantic province, where reefs (32.9%), sandy bottom (21.1%), and seagrass (19.7%) were the dominant habitats, and smaller contributions of rocky bottom, mangrove, and macroalgae were also found. The South Atlantic province had a lower variety of habitats associated with the Nurse Sharks, being composed of reefs and sandy and rocky bottoms (28.6% for each type) (Fig. 3B). Studies performed within MPAs had a greater abundance and diversity of habitat types than in locations outside these borders (Fig. 4).

The most reported abiotic variable in the retained studies (*N* = 30) was depth (73.3%), followed by temperature (23.3%), and salinity (13.3%). Populational data available in the studies (*N* = 30) were size (53.3%), description of breeding events (36.6%), and sex ratio (28.6%). The systems where the Atlantic Nurse Sharks were observed had a maximum depth of 75 m, temperatures ranging from 25 °C to 34 °C, and salinities varying from 31 to 38 ppt.

**Figure 5:**
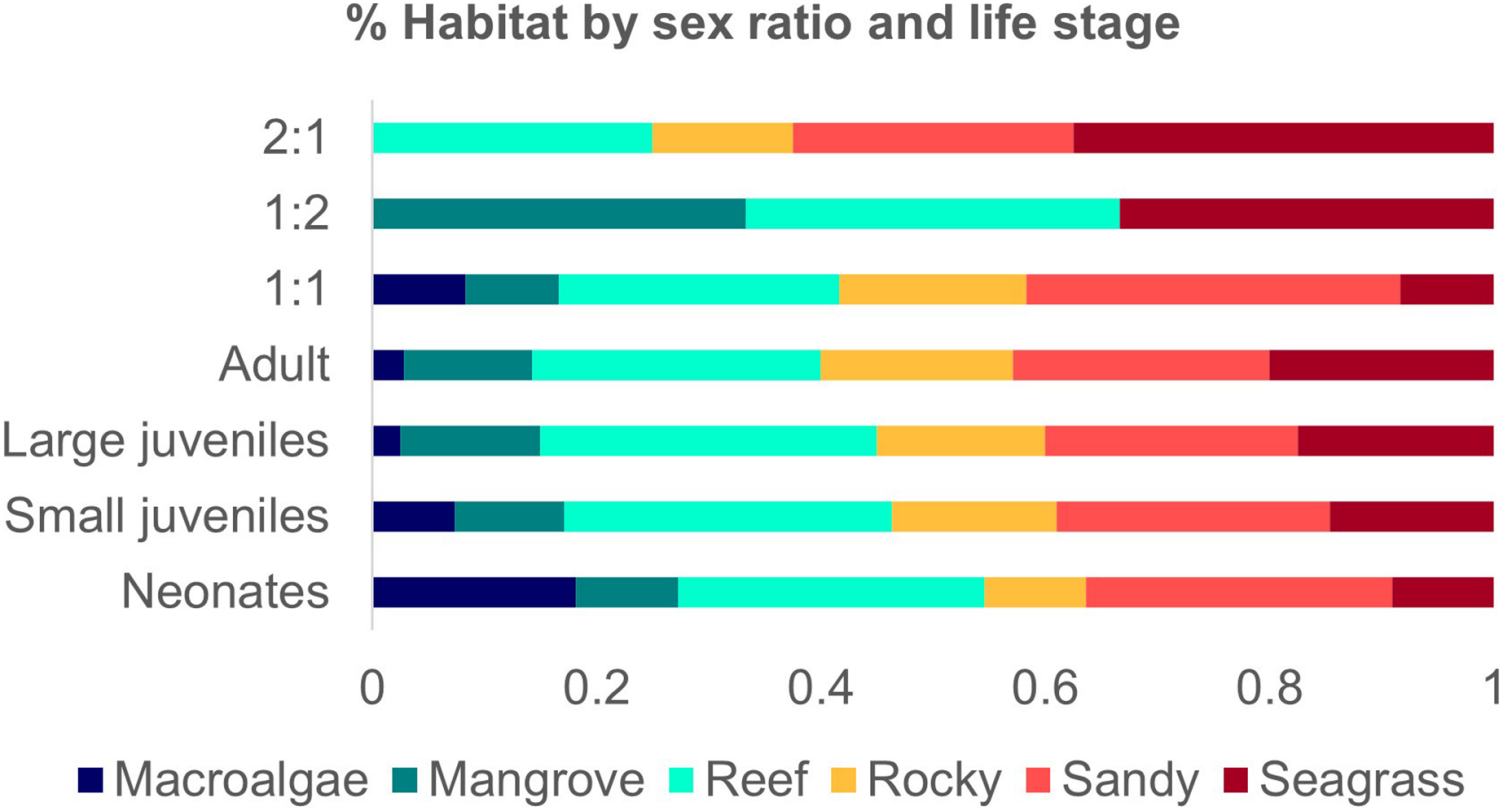
Number of studies of Atlantic Nurse Shark habitat types by sex ratio (A) and number of occurrences of Atlantic Nurse Sharks at habitat types by life stage classes (B).

The habitat of neonates was addressed in only two studies (Pratt & Carrier, 2007; Garla et al., 2009; Garla, Garrone-Neto & Gadig, 2014), with occurrences in reefs, rocky, macroalgae, sandy, and seagrass at a maximum depth of 20 m, with an average temperature of 26 °C and a salinity of 36 ppt. Adult sharks ranged from 215 to 380 TL cm and were recorded in all habitat categories (Castro, 2000; Chapman et al., 2005; Pratt & Carrier, 2007; DeAngelis et al., 2008; Hannan et al., 2012; Afonso, Andrade & Hazin, 2014; Shipley et al., 2018; Hansell et al., 2018; Tinari & Hammerschlag, 2021; Stoffers et al., 2021) (Fig. 5). The majority of studies did not discriminate variation in size and sex per type of habitat.

A total of 11 (36.6%) studies provided information on breeding events in the habitats described, 72.7% in shallow waters. Mating grounds were 90.9% of those locations and 63.4% were characterized as nursery grounds, most of them inside MPAs (90.9%). Neonates and small juveniles were associated with all six habitat types. Mating events were related only to seagrass, sandy bottoms, and/or macroalgae flats. No mention of the habitats’ relation with reproductive events was detected for Northeastern Brazil and Guianan ecoregions.

Studies that provided sex ratio or abundance of females and males were divided into three groups to address possible sex segregation of habitat use. The one female: one male ratio group occurred in all habitat types (1F:1.077−1.02M; Ferreira et al., 2013; Afonso, Andrade & Hazin, 2014; Hannan et al., 2012; Rangel, Hammerschlag & Moreira, 2021). The group 1:0.5 had higher occurrences in sandy and seagrass types but also occurred in reefs and rocky habitats (1F:0.43−0.56 M; Chapman et al., 2005; DeAngelis et al., 2008; Pratt et al., 2018). The third group of one female: two males occurred in mangroves, reefs, and seagrass (1F:1:2.05M; Wiley & Simpfendorfer, 2007) (Fig.5).

## Discussion

Our study consolidates information on the essential habitats associated with the occurrence of the Atlantic Nurse Shark populations. Our results can assist in the strategic (not opportunistic) creation of MPAs to protect this species which is necessary for the conservation of sharks, as highlighted by Baldi et al. (2017). Additionally, we emphasize herein the lack of studies on the habitat structural complexity that mediates the interaction of the Atlantic Nurse Shark with other species and with abiotic variables (beyond temperature and salinity). These are also key information for modeling studies that can allow us to foresee and identify critical regions for the conservation of this *‘‘Vulnerable’’* species, and to manage habitats that could collapse with Atlantic Nurse Sharks’ disappearance. We also provide a set of gaps that may serve as a road map for future studies.

Although research on the Atlantic Nurse Shark has increased in the last decade, knowledge of its ecology in the Eastern Atlantic and its habitat use patterns are still lacking in the scientific literature. Recently, evidence of a nursery area, mostly sandy substrate, was described for the Atlantic Nurse Shark and other shark species in Cabo Verde (Sal Rei Bay, Boa Vista Island), with sizes ranging from 43 to 140 cm (Rosa et al., 2023). Despite the sharks sampled in temperate realms on the first search, most studies (96.8%) were performed in Tropical Atlantic Waters. This may be related to the warmer temperatures found associated with Atlantic Nurse Shark abundance. The higher number of Atlantic Nurse Shark-habitat associations and diversity of habitats occurred in the Northwestern Atlantic, which comprehends the greatest expenditure in shark tourism along the Atlantic Nurse Shark distribution (Cisneros-Montemayor et al., 2013). These places are usually chosen precisely for the abundance of sharks (Gallagher et al., 2015) and, thus, enable higher research efforts. Therefore, it is important to highlight that our findings reflect the habitats associated with the Atlantic Nurse Sharks that were evaluated, not necessarily covering the entire range and diversity of habitats for the species, as studies focusing on its habitat use are still scarce. However, these findings are indicative of the habitats that harbor individuals and populations of the Atlantic Nurse Sharks and may be an indication for future studies to analyze habitat selection and correlate it with sex and ontogeny. Thus, it is possible that habitats of Atlantic Nurse Sharks that do not have the same visibility as tourist sites may be under greater impact but with less investment in research.

Sharks with larger sizes and occurrences in medium depths are more likely to be threatened due to lower population growth rates and refugees from fisheries (Dulvy et al., 2021). The Atlantic Nurse Shark is an example of a shallow-water shark that reaches up to 380 cm (Tinari & Hammerschlag, 2021). Thus, it is likely subjected to numerous coastal human impacts throughout its entire life, worsened by a lower ability to replace individuals removed from overfishing in populations, and a higher exposition to fisheries in shallow habitats (Dulvy et al., 2021). Considering that fishing pressure is an economic activity that already poses a great threat to chondrichthyans (Dulvy et al., 2021), our findings suggest that habitat loss could also be influencing the vulnerability of coastal populations. In addition, as most of the habitats used by *G. cirratum* are inserted in zones of high presence of touristic activities, it can be expected an even greater impact on the populations subjected to both habitat modification and behavior/movement influence. Considering the species’ recent change in the classification of extinction risk (from ‘*Data Deficient’* to *‘Vulnerable’*), new conservation plans should focus on mitigating the impacts of fishing and preserving the critical species’ habitat based on the data collected by this study to prevent further increase in extinction risk.

Besides the relationship between the Atlantic Nurse Shark populations and their essential habitats of occurrence, our findings could also provide insights into the ecological requirements of this species regarding water conditions. Populations were commonly found associated with shallow, coastal, warm, and saline waters in the studies retained herein. Even though these variables are important and may be an indicator of the species requirements, many other variables can be relevant in determining chondrichthyans’ occurrence. Visibility was not provided by the studies in this search, even though most of the study sites are recognized as high visibility areas (*e.g*., Glover’s Atoll, Bahamas, Florida Keys, Fernando de Noronha, Turks and Caicos, US Virgin Islands). Since Afonso, Andrade & Hazin, (2014) and Niella, Hazin & Afonso, (2017) related higher abundances with lower transparencies in Recife, Brazil, whether the Atlantic Nurse shark is dependent or not upon high transparency is yet to be revealed. Therefore, studies investigating the influence of visibility on the Atlantic Nurse Shark occurrence and other variables such as dissolved oxygen, hydrodynamics, and site isolation and connectivity, are still needed.

The IUCN Red List assessment of the Atlantic Nurse Shark indicates its occurrence in rocky, reefs, sandy, mangroves, and seagrasses habitats (Carlson et al., 2021), but our findings indicate reefs as the most important habitat for Atlantic Nurse Sharks. They represent at least 25% of the habitats encountered for all life stages, at both Northern and Southern Atlantic within and out of MPAs and were also described as nursery grounds. These habitats are known for providing refuge during the resting behavior on caves and crevices (Compagno, 2001) and are also the habitats where their main prey can be found in higher abundances (*i.e*., macroinvertebrates and small fish from the family Haemulidae; Castro, 2000). According to Carlson et al. (2021), the loss of coral reefs in Central America and the Southwestern Atlantic could be related to a decline in Atlantic Nurse Sharks’ observations. Coral reefs are known for their high diversity of species and structural complexity, supporting fishing stocks all over the world (Magel et al., 2019). Although Bascompte, Melian & Sala (2005) predicted top-down structuring of ecosystems with the Atlantic Nurse Shark as one of the top predators, studies have shown that reef-associated sharks occupy a mesopredator function (Roff et al., 2016; Desbiens et al., 2021). The destabilization generated by reef habitat degradation has effects that may scale up to the ecosystem level, leading to the loss of filtering services, oxygen depletion, and the extinction of commercially important fish and invertebrates (Worm et al., 2006). The effect of altered habitats and the relevance of habitat in maintaining species’ healthy has already been evidenced by Rangel, Hammerschlag & Moreira (2021), which showed lower nutritional quality in urban Atlantic Nurse Sharks compared to non-urban sharks. Thus, further studies should focus on the effects of the loss or degradation of specific habitats on the populational attributes of the Atlantic Nurse Sharks which are key to unveiling the patterns described in this study and can subsidize conservation measures with context-dependent information.

Although highly threatened by urbanization, deforestation, and grounding, mangroves are another example of a habitat that is important for several marine organisms, often acting as reproduction and recruitment zones. Neonates and small juveniles of Northwestern Atlantic populations of *G. cirratum* have been described to utilize mangroves as shelter during resting periods (Castro, 2000; Pratt & Carrier, 2007). However, no published data have investigated the use of this habitat by the Atlantic Nurse Sharks in the South Atlantic, even though this region harbors the world’s most extensive range of continuous mangroves, the Amazon Macrotidal Mangrove Coast (Souza-Filho, 2005). Seagrasses were also not mentioned in habitat studies in the Southern Atlantic for any life stages although associated with reproduction events in the Northwestern Atlantic (Carrier & Pratt, 1998; Wiley & Simpfendorfer, 2007; Pratt et al., 2018; Tinari & Hammerschlag, 2021; Pratt et al., 2022). The high fluvial discharge and consequent lower visibility may be one of the reasons seagrasses are not reported on the Northern coast of Brazil (Copertino, Creed & Lanari, 2016). However, there are hotspots for seagrass meadows on the Brazilian Northeast coast and sporadic occurrences on the southern distribution of Atlantic Nurse Sharks (Copertino, Creed & Lanari, 2016). Does this species not use these habitat types in Southern Waters or is there a gap in knowledge? Further studies would be required to address this question.

The occurrence of mainly females in sandy and seagrass habitats and the mating events described in these habitats might be related as some studies describe its use during breeding season as an avoidance strategy of males’ attempts to copulate or associated with thermoregulation during gestation (Pratt & Carrier, 2007; Afonso et al., 2016; Pratt et al., 2022). While males have been described by Pratt & Carrier (2007) to use deeper surrounding reefs as resting and staging areas. Studies in the Dry Tortugas have also described sex segregation in the use of space throughout the year, where males stay annually from May to July and females arrive biennially or triennially with peaks in June (when mating occurs) and September/August (Pratt & Carrier, 2007; Pratt et al., 2018; Pratt et al., 2022). Contrarily, in Recife, Northeastern Brazil, no pregnant female was captured, and the CPUE was lower from May to August with a seasonal pattern for males during the first quarter of the year (Ferreira et al., 2013). Further studies describing sex and size segregation in the use of habitat should be replicated enabling a complex understanding of possible habitat fidelity for some groups, philopatry, and allowing large-scale mapping of Atlantic Nurse Sharks’ movement and reproductive habitat use. Populational and genetic studies are also essential to assist in the definition of habitats to be protected considering their use as steppingstones in corridors between populations exchange. Until conducting more comprehensive and integrated research, we will remain unaware of the species’ dependence on specific habitats and their relationship to the site, as well as the possible influence of life stages that may trigger the periodic migration of individuals, such as reproduction in certain areas, and even refuge strategies that can increase the survival rate of neonates.

The Florida Keys, Dry Tortugas and Bahamas, USA, and Fernando de Noronha, Brazil are some localities known as breeding sites for the Atlantic Nurse Sharks (Fowler, 1906; Castro, 2000; Brooks et al., 2012; Afonso et al., 2016; Pratt et al., 2018). In agreement with Pratt et al. (2022), the Dry Tortugas is the breeding ground described in more detail for the Atlantic Nurse Shark. Although the authors describe characteristics shared with Fernando de Noronha and the Bahamas, such as the sand and seagrass flats, low-lying islands, high temperatures, and shallow waters near deep waters, it is still not well documented why this site is so important for the species’ reproduction (Pratt et al., 2022). Despite the increase in the number of studies in the last 14 years, it is still not possible to address the question proposed by Pratt & Carrier (2007): “What defines optimal habitat for Atlantic Nurse Shark’ neonates?”. Neonates were recorded in most habitats categorized here; however, all these data refer to only two localities (Fernando de Noronha, Brazil, and Dry Tortugas, USA) made by studies not directly focused on species-habitat relations. Thus, it is not possible to understand if the neonates actively select habitats that increase shelter against predation and not even if breeding events have taken place on these sites. Although the term “nursery grounds” was used in the studies to define areas with the presence of neonates and juveniles in consecutive years (Pratt & Carrier, 2007; Garla et al., 2009; Garla, Garrone-Neto & Gadig, 2014; Pratt et al., 2018), to define an area as a nursery, the place must meet the criteria proposed by Heupel, Carlson & Simpfendorfer (2007) which includes (1) sharks more commonly encountered in the area than other areas; (2) sharks with a tendency to remain or return for extended periods; and (3) the area or habitat is repeatedly used across years. Considering Heupel, Carlson & Simpfendorfer (2007) definition, Pratt et al. (2022) suggest that not only the Dry Tortugas is not used as a nursery but also that the Atlantic Nurse Shark does not use nurseries at all since the ovulation takes between 2–3 weeks (Castro, 2013) and consequently the release of fully developed pups could occur in different areas.

Overall, data on Atlantic Nurse Shark’s occurrence and habitat use, species interaction, and their interaction with abiotic conditions are still scarce throughout its entire distribution, but especially in the South Atlantic where the species is most threatened. Such information is critical while evaluating potential occurrence areas, as it can help identify satellite patches of specific habitats surrounding the study area that certain groups, like juveniles and pregnant females, might use. By incorporating this patchy perspective into their evaluations of the species and, potentially, protected areas, researchers and conservation agencies can gain a more comprehensive understanding of this species. Critical habitats for neonates and breeding sites are yet to be identified in Northeastern Brazil, and Guianan ecoregions. As highlighted by Pratt et al. (2022), breeding grounds are known to occur in remote areas, suggesting that anthropogenic development may have impacted coastal potential grounds. Considering that females seem to prefer shallow water during breeding, making them more vulnerable to fishing and other anthropogenic stressors (*e.g*., tourism, boat traffic, mooring, pollution, loss of mangroves ecosystems, and acoustic pollution; Carrier & Pratt, 1998; Arthington et al., 2016; Dulvy et al., 2021; Wosnick et al., 2021). The only known reproduction site for this species in the entire Southwestern Atlantic are the oceanic islands Fernando de Noronha and Rocas Atoll (Afonso et al., 2016). The study made by Karl, Castro & Garla (2012) showed through microsatellite analysis that Atlantic Nurse Shark populations from these islands were separated from the Brazilian coast highlighting the knowledge gaps on the reproductive events for a wide distribution of the species. In addition, studies are also needed to understand if the differences found in our study (*i.e*., no record for *G. cirratum* use of mangroves and seagrass in the South Atlantic) are due to a habitat selectivity of this species in the North and South Atlantic or due to poor sampling efforts in these regions.

The creation of new MPAs as long as the revision of the limits of the existing ones might both benefit the Atlantic Nurse Sharks and also be benefited from the increased knowledge of their requirements, considering their importance in ecosystem processes. Even though this process depends on the conservation status of some species, but also on politics and societal awareness, MPAs deliver the main conservation strategy to elasmobranchs through monitoring or prohibition of fishing (*i.e*., the main threat to this group) (Carlson et al., 2021; Dulvy et al., 2021). Our findings highlighted a role played by MPAs in protecting essential habitats since the greater diversity of habitats was found within their limits. According to Albano et al. (2021), MPAs are more effective for elasmobranchs when they include species-specific habitats of shark species, thus novel MPAs should consider our findings which highlight the importance of protecting shallow sandy and seagrass habitats mainly during the reproductive period and reef habitats for all life stages. The recent discovery of the Atlantic Nurse Shark occurrence in São Paulo, Brazil shows that it is still possible to find essential habitats where they occur and has been poorly explored by researchers (Santos et al., 2023). Currently, 7.9% of the Atlantic Nurse Shark occurrence area overlaps with MPAs, and the need for more numerous and larger MPAs (from tens to hundreds of km) encompassing a greater variety and connectivity between habitats are being evidenced (Chapman et al., 2005; Albano et al., 2021; Dwyer et al., 2020). A significant reduction in the Atlantic Nurse Shark demography at Rocas Atoll, Brazil, a no-take MPA, was predicted for the Intergovernmental Panel on Climate Change scenarios until 2100 (IPCC; Pörtner et al., 2019, Capitani et al., 2021). However, since the knowledge of the habitats required along the entire distribution of the Atlantic Nurse Sharks and their association with other water variables are still scarce, predictions for the future of climate change can underestimate the impacts on this species. Thus, understanding the ecological requirements, and the habitat structural complexity used by the different sex and size classes of the Atlantic Nurse Sharks is critical for defining optimal drivers for the species but also for trophic interactions associated with the habitats used.

## Conclusions

Conservation measures essential for the protection of the *‘Vulnerable’* Atlantic Nurse Shark include the creation of protected areas, fishing restriction measures, and closing access during the reproductive period. However, these measures rely on accurate scientific information that is still lacking for many populations of this species, thus it is crucial that a greater effort be undertaken in these regions so we may delineate efficient conservation measures. Otherwise, we would be spending valuable and limited resources in regions that do not encompass the living area of the target species making it difficult to protect *G. cirratum*, reduce mortality or restore its depleted populations (Dwyer et al., 2020). Further studies incorporating modeling techniques would also be useful to assist in the decision-making regarding this species and should incorporate habitat availability for the occurrence of Atlantic Nurse Sharks. Furthermore, studies that consider the future scenarios of tropicalization (increasing abundance of warm-affinity species) are also needed to understand new distribution ranges for the Atlantic Nurse Shark (McLean et al., 2021).

## Supplemental Information

10.7717/peerj.15540/supp-1 Supplemental Information 1 List of the studies obtained through a literature review of the distribution and habitat association of the Atlantic Nurse Shark *Ginglymostoma cirratum*.

10.7717/peerj.15540/supp-2 Supplemental Information 2 List of the studies obtained through a literature review of the habitat association of the Atlantic Nurse Shark *Ginglymostoma cirratum*.

## Additional Information and Declarations

### Data Availability

The following information was supplied regarding data availability: The lists of the studies obtained through a literature review of the distribution and habitat association of the Atlantic Nurse Shark *Ginglymostoma cirratum* are available in the Supplemental Files.
