# Supplementary material for: Habitat-use of the vulnerable Atlantic Nurse Shark: a review
Source: PeerJ. 2023 Jun 15;11:e15540. doi: 10.7717/peerj.15540 (PMC10276984; doi:10.7717/peerj.15540)
Supplement: Supplemental Information 1 [file peerj-11-15540-s001.docx]

**Habitat-use of the vulnerable Atlantic nurse shark: a review**

Vanessa Bettcher Brito^1,2^*, Ana Clara Sampaio Franco ^1,3^, Luciano Neves dos Santos^1,2,3^

^1^ Laboratório de Ictiologia Aplicada (LICTA), Universidade Federal do Estado do Rio de Janeiro (UNIRIO), Av. Pasteur, 458 – Sala 314A, CEP 22290-240, Rio de Janeiro, RJ, Brazil.

^2^ Programa de Pós-Graduação em Ecologia e Evolução (PPGEE), Universidade do Estado do Rio de Janeiro (UERJ), Rua São Francisco Xavier, 524, CEP 20550- 900, Rio de Janeiro, RJ, Brazil.

^3^ Programa de Pós-graduação em Biodiversidade Neotropical (PPGBIO), Universidade Federal do Estado do Rio de Janeiro (UNIRIO), Av. Pasteur, 458 – Sala 506A, CEP 22290-240, Rio de Janeiro, RJ, Brazil.

Corresponding Author:

Vanessa Bettcher^1^

Programa de Pós-Graduação em Ecologia e Evolução (PPGEE), Universidade do Estado do Rio de Janeiro (UERJ), Rua São Francisco Xavier, 524, CEP 20550- 900, Rio de Janeiro, RJ, Brazil.

Email address: vanessa_bettcher@hotmail.com

# Appendix 1 – List of the studies obtained through a literature review of the distribution and habitat association of the Atlantic Nurse Shark *Ginglymostoma cirratum* using as keywords "*Ginglymostoma cirratum*" OR "*Squalus punctulatus*" OR "*Squalus cirrhatus*" OR "*Ginglymostoma cirrhatum*" OR "*Squalus punctatus*" OR "*Scyllium cirrhosum*" OR "*Ginglymostoma cirrosum*" OR "*Squalus argus*" OR "*Ginglymostoma fulvum*" OR "*Ginglymostoma caboverdianus*" OR "*Ginglymostoma cirrotum*" at the Dimensions research database.

Adamson, M. L., & Caira, J. N. (1991). *Lockenloia sanguinis* n. gen., n. sp. (Nematoda: Dracunculoidea) from the heart of a nurse shark, *Ginglymostoma cirratum*, in Florida. Journal of Parasitology, 77(5), 663–665. https://doi.org/10.2307/3282695

Afonso, A. S., Andrade, H. A., & Hazin, F. H. V. (2014). Structure and Dynamics of the Shark Assemblage off Recife, Northeastern Brazil. PLOS ONE, 9(7), e102369. https://doi.org/10.1371/journal.pone.0102369

Afonso, A. S., Cantareli, C. V, Levy, R. P., & Veras, L. B. (2016). Evasive mating behaviour by female nurse sharks, *Ginglymostoma cirratum* (Bonnaterre, 1788), in an equatorial insular breeding ground. Neotropical Ichthyology, 14(4). https://doi.org/10.1590/1982-0224-20160103

Afonso, A. S., Hazin, F. H. V, Carvalho, F., Pacheco, J. C., Hazin, H., Kerstetter, D. W., et al. Burgess, G. H. (2011). Fishing gear modifications to reduce elasmobranch mortality in pelagic and bottom longline fisheries off Northeast Brazil. Fisheries Research, 108(2–3), 336–343. https://doi.org/10.1016/j.fishres.2011.01.007

Altobelli, A. N., & Szedlmayer, S. T. (2020). Migration and Residency of Sandbar, Atlantic Sharpnose, Bull, and Nurse Sharks in the Northern Gulf of Mexico. North American Journal of Fisheries Management, 40(5), 1324–1343. https://doi.org/10.1002/nafm.10501

Aragão, G., Pessoa, G., Kotas, J. E., & Spach, H. (2019). O CONHECIMENTO ECOLÓGICO LOCAL DOS PESCADORES ARTESANAIS SOBRE OS ELASMOBRÂNQUIOS MARINHO-COSTEIROS NA APA DO DELTA DO PARNAÍBA, NORDESTE DO BRASIL. Arquivos de Ciências Do Mar, 52(1), 34–49. https://doi.org/10.32360/acmar.v52i1.33667

Aragão, G. M. de O., Kotas, J. E., & Spach, H. L. (2020). UTILIZAÇÃO DE UMA ÁREA DE PROTEÇÃO AMBIENTAL POR UMA COMUNIDADE DE ELASMOBRÂNQUIOS NO ATLÂNTICO SUL OCIDENTAL. Boletim Do Laboratório de Hidrobiologia, 30(1). https://doi.org/10.18764/1981-6421e2020.1

AtallahBenson, L., Merly, L., Cray, C., & Hammerschlag, N. (2020). Serum Protein Analysis of Nurse Sharks. Journal of Aquatic Animal Health, 32(2), 77–82. https://doi.org/10.1002/aah.10100

Aucoin, S., Weege, S., Toebe, M., Guertin, J., Gorham, J., & Bresette, M. (2017). A new underwater shark capture method used by divers to catch and release nurse sharks (*Ginglymostoma cirratum*). Fishery Bulletin, 115(4), 484–495. https://doi.org/10.7755/fb.115.4.5

Aybar, L., Shin, D.-H., & Smith, S. L. (2009). Molecular characterization of the alpha subunit of complement component C8 (GcC8α) in the nurse shark (*Ginglymostoma cirratum*). Fish & Shellfish Immunology, 27(3), 397–406. https://doi.org/10.1016/j.fsi.2009.05.020

Bass, N. M., Manning, J. A., & Luer, C. A. (1991). Isolation and characterization of fatty acid binding protein in the liver of the nurse shark, *Ginglymostoma cirratum*. Comparative Biochemistry and Physiology Part A Molecular & Integrative Physiology, 98(2), 355–362. https://doi.org/10.1016/0300-9629(91)90547-p

Bodine, A. B., Luer, C. A., & Gangjee, S. (1984). Determination of ceruloplasmin and other copper transport ligands in the blood sera of the nurse shark and clearnose skate. Comparative Biochemistry and Physiology Part B Comparative Biochemistry, 77(4), 779–783. https://doi.org/10.1016/0305-0491(84)90312-2

Bodine, A. B., Luer, C. A., & Gangjee, S. (1985). A comparative study of monooxygenase activity in elasmobranchs and mammals: Activation of the model pro-carcinogen aflatoxin B1 by liver preparations of calf, nurse shark and clearnose skate. Comparative Biochemistry and Physiology Part C Toxicology & Pharmacology, 82(2), 255–257. https://doi.org/10.1016/0742-8413(85)90159-8

Borucinska, J., & Caira, J. N. (1993). A comparison of mode of attachment and histopathogenicity of four tapeworm species representing two orders infecting the spiral intestine of the nurse shark, *Ginglymostoma cirratum*. Journal of Parasitology, 79(2), 238–246. https://doi.org/10.2307/3283514

Bouyoucos, I. A., Talwar, B. S., Brooks, E. J., Brownscombe, J. W., Cooke, S. J., Suski, C. D., & Mandelman, J. W. (2018). Exercise intensity while hooked is associated with physiological status of longline-captured sharks. Conservation Physiology, 6(1), coy074. https://doi.org/10.1093/conphys/coy074

Bruns, S., & Henderson, A. C. (2021). A baited remote underwater video system (BRUVS) assessment of elasmobranch diversity and abundance on the eastern Caicos Bank (Turks and Caicos Islands); an environment in transition. Environmental Biology of Fishes, 103(9), 1001–1012. https://doi.org/10.1007/s10641-020-01004-4

Buteau, G. H., Simmons, J. E., & Fairbairn, D. (1969). Lipid metabolism in helminth parasites IX. Fatty acid composition of shark tapeworms and of their hosts. Experimental Parasitology, 26(2), 209–213. https://doi.org/10.1016/0014-4894(69)90114-3

Caballero, S., Galeano, A. M., Lozano, J. D., & Vives, M. (2020). Description of the microbiota in epidermal mucus and skin of sharks (*Ginglymostoma cirratum* and Negaprion brevirostris) and one stingray (Hypanus americanus). PeerJ, 8, e10240. https://doi.org/10.7717/peerj.10240

Caira, J. N., & Jolitz, E. C. (1989). Gut pH in the Nurse Shark, *Ginglymostoma cirratum* (Bonnaterre). Copeia, 1989(1), 192. https://doi.org/10.2307/1445621

Carrier, J. C., & Luer, C. A. (1990). Growth Rates in The Nurse Shark, *Ginglymostoma cirratum*. Copeia, 1990(3), 686. https://doi.org/10.2307/1446435

Carrier, J. C., & Pratt, H. L. (1998). Habitat management and closure of a nurse shark breeding and nursery ground. Fisheries Research, 39(2), 209–213. https://doi.org/10.1016/s0165-7836(98)00184-2

Carrier, J. C., Murru, F. L., Walsh, M. T., & Pratt, H. L. (2003). Assessing reproductive potential and gestation in nurse sharks (*Ginglymostoma cirratum*) using ultrasonography and endoscopy: An example of bridging the gap between field research and captive studies. Zoo Biology, 22(2), 179–187. https://doi.org/10.1002/zoo.10088

Carrier, J. C., Pratt, H. L., & Martin, L. K. (1994). Group Reproductive Behaviors in Free-Living Nurse Sharks, *Ginglymostoma cirratum*. Copeia, 1994(3), 646. https://doi.org/10.2307/1447180

Casper, B. M., & Mann, D. A. (2006). Evoked Potential Audiograms of the Nurse Shark (*Ginglymostoma cirratum*) and the Yellow Stingray (Urobatis jamaicensis). Environmental Biology of Fishes, 76(1), 101–108. https://doi.org/10.1007/s10641-006-9012-9

Casper, B. M., & Mann, D. A. (2009). Field hearing measurements of the Atlantic sharpnose shark Rhizoprionodon terraenovae. Journal of Fish Biology, 75(10), 2768–2776. https://doi.org/10.1111/j.1095-8649.2009.02477.x

Castro, A. L. F., & Rosa, R. S. (2005). Use of natural marks on population estimates of the nurse shark,*Ginglymostoma cirratum*, at Atol das Rocas Biological Reserve, Brazil. Environmental Biology of Fishes, 72(2), 213–221. https://doi.org/10.1007/s10641-004-1479-7

Castro, J. I. (2000). The Biology of the Nurse Shark, *Ginglymostoma cirratum*, Off the Florida East Coast and the Bahama Islands. Environmental Biology of Fishes, 58(1), 1–22. https://doi.org/10.1023/a:1007698017645

Chapman, D. D., Pikitch, E. K., Babcock, E., & Shivji, M. S. (2005). Marine Reserve Design and Evaluation Using Automated Acoustic Telemetry: A Case-study Involving Coral Reef-associated Sharks in the Mesoamerican Caribbean. Marine Technology Society Journal, 39(1), 42–55. https://doi.org/10.4031/002533205787521640

Clem, L. W., & Leslie, G. A. (1971). Production of 19S IgM Antibodies with Restricted Heterogeneity from Sharks. Proceedings of the National Academy of Sciences of the United States of America, 68(1), 139–141. https://doi.org/10.1073/pnas.68.1.139

Credille, K. M., Johnson, L. K., & Reimschuessel, R. (1993). Parasitic Meningoencephalitis in Nurse Sharks (*Ginglymostoma cirratum*). Journal of Wildlife Diseases, 29(3), 502–506. https://doi.org/10.7589/0090-3558-29.3.502

Criscitiello, M. F., Kraev, I., & Lange, S. (2019). Deiminated proteins in extracellular vesicles and plasma of nurse shark (*Ginglymostoma cirratum*) - Novel insights into shark immunity. Fish & Shellfish Immunology, 92, 249–255. https://doi.org/10.1016/j.fsi.2019.06.012

Cserr, H. F., & Ostrach, L. H. (1974). On the presence of subarachnoid fluid in the mudpuppy, Necturus maculosus. Comparative Biochemistry and Physiology Part A Molecular & Integrative Physiology, 48(1), 145–151. https://doi.org/10.1016/0300-9629(74)90862-7

de Sousa Rangel, B., Hammerschlag, N., & Moreira, R. G. (2021). Urban living influences the nutritional quality of a juvenile shark species. The Science of The Total Environment, 776, 146025. https://doi.org/10.1016/j.scitotenv.2021.146025

Deiss, T. C., Breaux, B., Ott, J. A., Daniel, R. A., Chen, P. L., Castro, C. D., et al. Criscitiello, M. F. (2019). Ancient Use of Ig Variable Domains Contributes Significantly to the TCRδ Repertoire. The Journal of Immunology, 203(5), 1265–1275. https://doi.org/10.4049/jimmunol.1900369

Díaz, M. JM. (2016). CONSIDERACIONES ZOOGEOGRAFIAS SOBRE LOS TIBURONES DEL PACIFICO COLOMBIANO. Boletín de Investigaciones Marinas y Costeras, 13. https://doi.org/10.25268/bimc.invemar.1983.13.0.482

Dillon, E. M., McCauley, D. J., Morales-Saldaña, J. M., Leonard, N. D., Zhao, J., & O’Dea, A. (2021). Fossil dermal denticles reveal the preexploitation baseline of a Caribbean coral reef shark community. Proceedings of the National Academy of Sciences of the United States of America, 118(29), e2017735118. https://doi.org/10.1073/pnas.2017735118

Dwyer, R. G., Krueck, N. C., Udyawer, V., Heupel, M. R., Chapman, D., Pratt, H. L., et al. Simpfendorfer, C. A. (2020). Individual and Population Benefits of Marine Reserves for Reef Sharks. Current Biology, 30(3), 480-489.e5. https://doi.org/10.1016/j.cub.2019.12.005

Ebbesson, S. O. E., & Heimer, L. (1970). Projections of the olfactory tract fibers in the nurse shark (*Ginglymostoma cirratum*). Brain Research, 17(1), 47–55. https://doi.org/10.1016/0006-8993(70)90307-0

FÄNGE, R., & MATTISSON, A. (1981). THE LYMPHOMYELOID (HEMOPOIETIC) SYSTEM OF THE ATLANTIC NURSE SHARK, *GINGLYMOSTOMA CIRRATUM*. Biological Bulletin, 160(2), 240–249. https://doi.org/10.2307/1540884

Ferreira, L. C., Afonso, A. S., Castilho, P. C., & Hazin, F. H. V. (2013). Habitat use of the nurse shark, *Ginglymostoma cirratum*, off Recife, Northeast Brazil: a combined survey with longline and acoustic telemetry. Environmental Biology of Fishes, 96(6), 735–745. https://doi.org/10.1007/s10641-012-0067-5

Gallagher, A. J., Staaterman, E. R., Cooke, S. J., & Hammerschlag, N. (2017). Behavioural responses to fisheries capture among sharks caught using experimental fishery gear. Canadian Journal of Fisheries and Aquatic Sciences, 74(1), 1–7. https://doi.org/10.1139/cjfas-2016-0165

Garla, R. C., Gadig, O. B. F., & Garrone-Neto, D. (2017). Movement and activity patterns of the nurse shark, *Ginglymostoma cirratum*, in an oceanic Marine Protected Area of the South-western Atlantic. Journal of the Marine Biological Association of the United Kingdom, 97(8), 1565–1572. https://doi.org/10.1017/s0025315416001028

Garla, R. C., Garcia, J., Veras, L. B., & Lopes, N. P. (2009). Fernando de Noronha as an insular nursery area for lemon sharks, Negaprion brevirostris, and nurse sharks, *Ginglymostoma cirratum*, in the equatorial western Atlantic Ocean. Marine Biodiversity Records, 2. https://doi.org/10.1017/s1755267209000670

Garla, R. C., Garrone-Neto, D., & Gadig, O. B. F. (2014). Defensive strategies of neonate nurse sharks, *Ginglymostoma cirratum*, in an oceanic archipelago of the Western Central Atlantic. Acta Ethologica, 18(2), 167–171. https://doi.org/10.1007/s10211-014-0200-x

Gerald, K. B., Matis, J. H., & Kleerekoper, H. (1978). A stochastic locomotor control model for the nurse shark, *Ginglymostoma cirratum*. Journal of Mathematical Biology, 6(1), 37–48. https://doi.org/10.1007/bf02478515

Gilbert, P. W., Sivak, J. G., & Pelham, R. E. (1981). Rapid pupil change in selachians. Canadian Journal of Zoology, 59(3), 560–564. https://doi.org/10.1139/z81-081

Goldstein, L., & Dewitt-Harley, S. (1973). Trimethylamine oxidase of nurse shark liver and its relation to mammalian mixed function amine oxidase. Comparative Biochemistry and Physiology Part B Comparative Biochemistry, 45(4), 895–903. https://doi.org/10.1016/0305-0491(73)90150-8

Goldstein, L., & Funkhouser, D. (1972). Biosynthesis of trimethylamine oxide in the nurse shark, *Ginglymostoma cirratum*. Comparative Biochemistry and Physiology Part A Molecular & Integrative Physiology, 42(1), 51–57. https://doi.org/10.1016/0300-9629(72)90365-9

Gore, M., Ormond, R., Clarke, C., Kohler, J., Millar, C., & Brooks, E. (2020). Application of Photo-Identification and Lengthened Deployment Periods to Baited Remote Underwater Video Stations (BRUVS) Abundance Estimates of Coral Reef Sharks. Oceans, 1(4), 274–299. https://doi.org/10.3390/oceans1040019

Gutowsky, L. F. G., Rider, M. J., Roemer, R. P., Gallagher, A. J., Heithaus, M. R., Cooke, S. J., & Hammerschlag, N. (2021). Large sharks exhibit varying behavioral responses to major hurricanes. Estuarine Coastal and Shelf Science, 256, 107373. https://doi.org/10.1016/j.ecss.2021.107373

Guttridge, T. L., Gruber, S. H., Gledhill, K. S., Croft, D. P., Sims, D. W., & Krause, J. (2009). Social preferences of juvenile lemon sharks, Negaprion brevirostris. Animal Behaviour, 78(2), 543–548. https://doi.org/10.1016/j.anbehav.2009.06.009

Hannan, K. M., Driggers, W. B., Hanisko, D. S., Jones, L. M., & Canning, A. B. (2012). Distribution of the Nurse Shark, *Ginglymostoma cirratum*, in the Northern Gulf of Mexico. Bulletin of Marine Science, 88(1), 73–80. https://doi.org/10.5343/bms.2011.1033

Hansell, A. C., Kessel, S. T., Brewster, L. R., Cadrin, S. X., Gruber, S. H., Skomal, G. B., & Guttridge, T. L. (2018). Local indicators of abundance and demographics for the coastal shark assemblage of Bimini, Bahamas. Fisheries Research, 197, 34–44. https://doi.org/10.1016/j.fishres.2017.09.016

Heist, E. J., Carrier, J. C., Pratt, H. L., & Pratt, T. C. (2011). Exact Enumeration of Sires in the Polyandrous Nurse Shark (*Ginglymostoma cirratum*). Copeia, 2011(4), 539–544. https://doi.org/10.1643/ce-10-165

Heist, E. J., Jenkot, J. L., Keeney, D. B., Lane, R. L., Moyer, G. R., Reading, B. J., & Smith, N. L. (2003). Isolation and characterization of polymorphic microsatellite loci in nurse shark (*Ginglymostoma cirratum*). Molecular Ecology Resources, 3(1), 59–61. https://doi.org/10.1046/j.1471-8286.2003.00348.x

Hendon, J. M., Hoffmayer, E. R., & Driggers, W. B. (2013). First Record of a Nurse Shark, *Ginglymostoma cirratum*, within the Mississippi Sound. Gulf and Caribbean Research, 25. https://doi.org/10.18785/gcr.2501.13

Irschick, D. J., & Hammerschlag, N. (2014). A new metric for measuring condition in large predatory sharks. Journal of Fish Biology, 85(3), 917–926. https://doi.org/10.1111/jfb.12484

Jensen, J. A. (1969). A SPECIFIC INACTIVATOR OF MAMMALIAN C&#x27;4 ISOLATED FROM NURSE SHARK (*Ginglymostoma cirratum*) SERUM. Journal of Experimental Medicine, 130(2), 217–241. https://doi.org/10.1084/jem.130.2.217

Jerome, J. M., Gallagher, A. J., Cooke, S. J., & Hammerschlag, N. (2018). Integrating reflexes with physiological measures to evaluate coastal shark stress response to capture. ICES Journal of Marine Science, 75(2), 796–804. https://doi.org/10.1093/icesjms/fsx191

Johnson, C. S., Scronce, B. L., & McManus, M. W. (1984). Detection of DC electric dipoles in background fields by the nurse shark. Journal of Comparative Physiology A, 155(5), 681–687. https://doi.org/10.1007/bf00610854

Karl, S. A., Castro, A. L. F., & Garla, R. C. (2012). Population genetics of the nurse shark (*Ginglymostoma cirratum*) in the western Atlantic. Marine Biology, 159(3), 489–498. https://doi.org/10.1007/s00227-011-1828-y

Kashiwagi, T., Kingsland, K. M., Pratt, T. C., Pratt, H. L., & Heist, E. J. (2016). Complete mitochondrial genome of the nurse shark *Ginglymostoma cirratum*. Mitochondrial DNA Part B, 1(1), 464–465. https://doi.org/10.1080/23802359.2016.1186514

Kendall, C., Valentino, S., Bodine, A. B., & Luer, C. A. (1994). Triploidy in a Nurse Shark, *Ginglymostoma cirratum*. Copeia, 1994(3), 825. https://doi.org/10.2307/1447205

Kleerekoper, H., Gruber, D., & Matis, J. (1975). Accuracy of localization of a chemical stimulus in flowing and stagnant water by the nurse shark,*Ginglymostoma cirratum*. Journal of Comparative Physiology A, 98(3), 257–275. https://doi.org/10.1007/bf00656973

Lear, K. O., Whitney, N. M., Brewster, L. R., Morris, J. J., Hueter, R. E., & Gleiss, A. C. (2016). Correlations of metabolic rate and body acceleration in three species of coastal sharks under contrasting temperature regimes. Journal of Experimental Biology, 220(3), 397–407. https://doi.org/10.1242/jeb.146993

Lennon, E., and Sealey, K. S. (2021). Elasmobranch (Chondrichthyes, Elasmobranchii) Habitat Use in an Insular Tropical Lagoon in Exuma, The Bahamas. Caribbean Journal of Science 51(1): 20-29. https://doi.org/10.18475/cjos.v51i1.a3

Luer, C. A., Blum, P. C., & Gilbert, P. W. (1990). Rate of Tooth Replacement in the Nurse Shark, *Ginglymostoma cirratum*. Copeia, 1990(1), 182. https://doi.org/10.2307/1445834

Luiten, P. G. M. (1981). Two visual pathways to the telencephalon in the nurse shark (*Ginglymostoma cirratum*). I. Retinal projections. The Journal of Comparative Neurology, 196(4), 531–538. https://doi.org/10.1002/cne.901960402

Luiten, P. G. M. (1981). Two visual pathways to the telencephalon in the nurse shark (*Ginglymostoma cirratum*). II. Ascending thalamo‐telencephalic connections. The Journal of Comparative Neurology, 196(4), 539–548. https://doi.org/10.1002/cne.901960403

Lundblad, G., Fänge, R., & Slettengren, K. (1983). β-N-Acetylhexosaminidase and α-d-mannosidase from the epigonal lymphomyeloid organ of the nurse shark (*Ginglymostoma cirratum*). Comparative Biochemistry and Physiology Part B Comparative Biochemistry, 76(2), 277–281. https://doi.org/10.1016/0305-0491(83)90070-6

Martínez-Candelas, I. A., Pérez-Jiménez, J. C., Espinoza-Tenorio, A., McClenachan, L., & Méndez-Loeza, I. (2020). Use of historical data to assess changes in the vulnerability of sharks. Fisheries Research, 226, 105526. https://doi.org/10.1016/j.fishres.2020.105526

Mathewson, R. F., & Hodgson, E. S. (1972). Klinotaxis and rheotaxis in orientation of sharks toward chemical stimuli. Comparative Biochemistry and Physiology Part A Molecular & Integrative Physiology, 42(1), 79–84. https://doi.org/10.1016/0300-9629(72)90369-6

Matis, J., Kleerekoper, H., & Childers, D. (1974). Cycles in anilmals. Biological Rhythm Research, 5(3–4), 259–266. https://doi.org/10.1080/09291017409359434

Matis, J. H., Kleerekoper, H., & Gruber, D. (1975). The logomotor behavior of the nurse shark,*Ginglymostoma cirratum*; A time series analysis. Acta Biotheoretica, 24(3–4), 127–135. https://doi.org/10.1007/bf01557000

McClung, J. R., Gelderd, J. B., & Whitman, R. A. (1978). Axosomatic synapses in the spinal cord of the shark. Experimental Neurology, 61(3), 609–614. https://doi.org/10.1016/0014-4886(78)90027-4

Moorhead, S. G., Gallagher, A. J., Merly, L., & Hammerschlag, N. (2021). Variation of body condition and plasma energy substrates with life stage, sex, and season in wild‐sampled nurse sharks *Ginglymostoma cirratum*. Journal of Fish Biology, 98(3), 680–693. https://doi.org/10.1111/jfb.14612

Motta, P. J., & Wilga, C. D. (1999). Anatomy of the feeding apparatus of the nurse shark, *Ginglymostoma cirratum*. Journal of Morphology, 241(1), 33–60. https://doi.org/10.1002/(sici)1097-4687(199907)241:1<33::aid-jmor3>3.0.co;2-1

Motta, P. J., Hueter, R. E., Tricas, T. C., & Summers, A. P. (2002). Kinematic Analysis of Suction Feeding in the Nurse Shark, *Ginglymostoma cirratum* (Orectolobiformes, Ginglymostomatidae). Copeia, 2002(1), 24–38. https://doi.org/10.1643/0045-8511(2002)002[0024:kaosfi]2.0.co;2

Motta, P. J., Hueter, R. E., Tricas, T. C., Summers, A. P., Huber, D. R., Lowry, D., et al. Wintzer, A. P. (2008). Functional morphology of the feeding apparatus, feeding constraints, and suction performance in the nurse shark *Ginglymostoma cirratum*. Journal of Morphology, 269(9), 1041–1055. https://doi.org/10.1002/jmor.10626

Nascimento, D. M., Alves, R. R. N., Barboza, R. R. D., Schmidt, A. J., Diele, K., & Mourão, J. S. (2017). Commercial relationships between intermediaries and harvesters of the mangrove crab Ucides cordatus (Linnaeus, 1763) in the Mamanguape River estuary, Brazil, and their socio-ecological implications. Ecological Economics, 131, 44–51. https://doi.org/10.1016/j.ecolecon.2016.08.017

Niella, Y. V, Hazin, F. H. V, & Afonso, A. S. (2017). Detecting Multispecific Patterns in the Catch Composition of a Fisheries‐Independent Longline Survey. Marine and Coastal Fisheries, 9(1), 388–395. https://doi.org/10.1080/19425120.2017.1347115

O’Connell, C. P., & He, P. (2014). A large scale field analysis examining the effect of magnetically-treated baits and barriers on teleost and elasmobranch behavior. Ocean & Coastal Management, 96, 130–137. https://doi.org/10.1016/j.ocecoaman.2014.05.011

O’Connell, C. P., Abel, D. C., Rice, P. H., Stroud, E. M., & Simuro, N. C. (2010). Responses of the southern stingray (Dasyatis americana) and the nurse shark (*Ginglymostoma cirratum*) to permanent magnets. Marine and Freshwater Behaviour and Physiology, 43(1), 63–73. https://doi.org/10.1080/10236241003672230

Ohta, Y., McKinney, E. C., Criscitiello, M. F., & Flajnik, M. F. (2002). Proteasome, Transporter Associated with Antigen Processing, and Class I Genes in the Nurse Shark *Ginglymostoma cirratum*: Evidence for a Stable Class I Region and MHC Haplotype Lineages. The Journal of Immunology, 168(2), 771–781. https://doi.org/10.4049/jimmunol.168.2.771

Ohta, Y., Landis, E., Boulay, T., Phillips, R. B., Collet, B., Secombes, C. J., et al. Hansen, J. D. (2004). Homologs of CD83 from Elasmobranch and Teleost Fish. The Journal of Immunology, 173(7), 4553–4560. https://doi.org/10.4049/jimmunol.173.7.4553

Olsson, Y., Carsten, A. L., & Klatzo, I. (1972). Effects of gamma radiation on the shark brain. Acta Neuropathologica, 21(1), 1–10. https://doi.org/10.1007/bf00687995

Oppelt, W. W., Adamson, R. H., Zubrod, C. G., & Rall, D. P. (1966). Further observations on the physiology and pharmacology of elasmobranch ventricular fluid. Comparative Biochemistry and Physiology, 17(3), 857–866. https://doi.org/10.1016/0010-406x(66)90126-5

Oppelt, W. W., Patlak, C. S., Zubrod, C. G., & Rall, D. P. (1964). Ventricular fluid production rates and turnover in elasmobranchii. Comparative Biochemistry and Physiology, 12(2), 171–177. https://doi.org/10.1016/0010-406x(64)90171-9

Pratt, H. L., & Carrier, J. C. (2001). A Review of Elasmobranch Reproductive Behavior with a Case Study on the Nurse Shark, *Ginglymostoma cirratum*. Environmental Biology of Fishes, 60(1–3), 157–188. https://doi.org/10.1023/a:1007656126281

Pratt, H. L., Pratt, T. C., Morley, D., Lowerre-Barbieri, S., Collins, A., Carrier, J. C., et al. Whitney, N. M. (2018). Partial migration of the nurse shark, *Ginglymostoma cirratum* (Bonnaterre), from the Dry Tortugas Islands. Environmental Biology of Fishes, 101(4), 515–530. https://doi.org/10.1007/s10641-017-0711-1

Rada, D. P., Burgess, G. H., Rosa, R. S., & Gadig, O. F. (2014). Necrophagy of a nurse shark (*Ginglymostoma cirratum*) by tiger sharks (Galeocerdo cuvier). Universitas Scientiarum, 20(3), 313–320. https://doi.org/10.11144/javeriana.sc20-3.noan

Rangel, B. S., Hammerschlag, N., Sulikowski, J. A., & Moreira, R. G. (2021). Physiological markers suggest energetic and nutritional adjustments in male sharks linked to reproduction. Oecologia, 196(4), 989–1004. https://doi.org/10.1007/s00442-021-04999-4

Rêgo, M. G., Araujo, M. L. G., Barros, M. E. G., Aires, L. D., Oliveira, P. G. V, Hazin, F. H. V, et al. Evêncio-Neto, J. (2019). Morphological description of ovary and uterus of the nurse shark (*Ginglymostoma cirratum*) caught off at the Fortaleza coast, Northeast Brazil. Pesquisa Veterinária Brasileira, 39(12), 997–1004. https://doi.org/10.1590/1678-5150-pvb-6141

Rêgo, M. G., Fitzpatrick, J., Hazin, F. H. V, de Araújo, M. L. G., da Silveira, L. M., Oliveira, P. G. V, & Evêncio-Neto, J. (2015). Characterization of testicular morphology and spermatogenesis in the nurse sharks *Ginglymostoma cirratum* (Bonnaterre, 1788). Zoomorphology, 134(1), 117–123. https://doi.org/10.1007/s00435-014-0240-9

Rumfelt, L. L., Avila, D., Diaz, M., Bartl, S., McKinney, E. C., & Flajnik, M. F. (2001). A shark antibody heavy chain encoded by a nonsomatically rearranged VDJ is preferentially expressed in early development and is convergent with mammalian IgG. Proceedings of the National Academy of Sciences of the United States of America, 98(4), 1775–1780. https://doi.org/10.1073/pnas.98.4.1775

Rumfelt, L. L., Diaz, M., Lohr, R. L., Mochon, E., & Flajnik, M. F. (2004). Unprecedented Multiplicity of Ig Transmembrane and Secretory mRNA Forms in the Cartilaginous Fish. The Journal of Immunology, 173(2), 1129–1139. https://doi.org/10.4049/jimmunol.173.2.1129

RUMFELT, L. L., McKINNEY, E. C., TAYLOR, E., & FLAJNIK, M. F. (2002). The Development of Primary and Secondary Lymphoid Tissues in the Nurse Shark *Ginglymostoma cirratum*: B‐Cell Zones Precede Dendritic Cell Immigration and T‐Cell Zone Formation During Ontogeny of the Spleen. Scandinavian Journal of Immunology, 56(2), 130–148. https://doi.org/10.1046/j.1365-3083.2002.01116.x

Sadowsky, V. (1967). Selachier aus dem Litoral von Sao Paulo, Brasilien. Beitrage Zur Neotropischen Fauna, 5(2), 71–88. https://doi.org/10.1080/01650526709360398

Santander-Neto, J., Shinozaki-Mendes, R. A., Silveira, L. M., Jucá-Queiroz, B., Furtado-Neto, M. A. A., & Faria, V. V. (2011). Population structure of nurse sharks, *Ginglymostoma cirratum* (Orectolobiformes), caught off Ceará State, Brazil, south-western Equatorial Atlantic. Journal of the Marine Biological Association of the United Kingdom, 91(6), 1193–1196. https://doi.org/10.1017/s0025315410001293

Saville, K. J., Lindley, A. M., Maries, E. G., Carrier, J. C., & Pratt, H. L. (2002). Multiple Paternity in the Nurse Shark, *Ginglymostoma cirratum*. Environmental Biology of Fishes, 63(3), 347–351. https://doi.org/10.1023/a:1014369011709

Sequeda, J. V., Sánchez, C. M. D., Campo, K. G., Londoño, T. L., Ruiz, M. D., & Gómez-López, D. I. (2016). BIODIVERSIDAD MARINA EN BAJO NUEVO, BAJO ALICIA Y BANCO SERRANILLA, RESERVA DE BIOSFERA SEAFLOWER. Boletín de Investigaciones Marinas y Costeras, 44(1). https://doi.org/10.25268/bimc.invemar.2015.44.1.27

Shipley, O. N., Murchie, K. J., Frisk, M. G., O’Shea, O. R., Winchester, M. M., Brooks, E. J., et al. Power, M. (2018). Trophic niche dynamics of three nearshore benthic predators in The Bahamas. Hydrobiologia, 813(1), 177–188. https://doi.org/10.1007/s10750-018-3523-1

Stoffers, T., de Graaf, M., Winter, H. V, & Nagelkerke, L. A. J. (2021). Distribution and ontogenetic habitat shifts of reef associated shark species in the northeastern Caribbean. Marine Ecology Progress Series, 665, 145–158. https://doi.org/10.3354/meps13688

Tilley, A., López-Angarita, J., & Turner, J. R. (2013). Diet Reconstruction and Resource Partitioning of a Caribbean Marine Mesopredator Using Stable Isotope Bayesian Modelling. PLOS ONE, 8(11), e79560. https://doi.org/10.1371/journal.pone.0079560

Tinari, A. M., & Hammerschlag, N. (2021). An ecological assessment of large coastal shark communities in South Florida. Ocean & Coastal Management, 211, 105772. https://doi.org/10.1016/j.ocecoaman.2021.105772

Trape, S. (2008). Shark Attacks in Dakar and the Cap Vert Peninsula, Senegal: Low Incidence despite High Occurrence of Potentially Dangerous Species. PLOS ONE, 3(1), e1495. https://doi.org/10.1371/journal.pone.0001495

Walsh, C. J., Toranto, J. D., Gilliland, C. T., Noyes, D. R., Bodine, A. B., & Luer, C. A. (2006). Nitric oxide production by nurse shark (*Ginglymostoma cirratum*) and clearnose skate (Raja eglanteria) peripheral blood leucocytes. Fish & Shellfish Immunology, 20(1), 40–46. https://doi.org/10.1016/j.fsi.2005.03.011

Whitney, N. M., Lear, K. O., Gaskins, L. C., & Gleiss, A. C. (2016). The effects of temperature and swimming speed on the metabolic rate of the nurse shark (*Ginglymostoma cirratum*, Bonaterre). Journal of Experimental Marine Biology and Ecology, 477, 40–46. https://doi.org/10.1016/j.jembe.2015.12.009

Wosnick, N., Chaves, A. P., Leite, R. D., Nunes, J. L. S., Saint’Pierre, T. D., Willmer, I. Q., & Hauser-Davis, R. A. (2021). Nurse sharks, space rockets and cargo ships: Metals and oxidative stress in a benthic, resident and large-sized mesopredator, *Ginglymostoma cirratum*. Environmental Pollution, 288, 117784. https://doi.org/10.1016/j.envpol.2021.117784
