# Supplementary material for: Habitat-use of the vulnerable Atlantic Nurse Shark: a review
Source: PeerJ. 2023 Jun 15;11:e15540. doi: 10.7717/peerj.15540 (PMC10276984; doi:10.7717/peerj.15540)
Supplement: Supplemental Information 2 [file peerj-11-15540-s002.docx]

**Habitat-use of the vulnerable Atlantic nurse shark: a review**

Vanessa Bettcher Brito^1,2^*, Ana Clara Sampaio Franco ^1,3^, Luciano Neves dos Santos^1,2,3^

^1^ Laboratório de Ictiologia Aplicada (LICTA), Universidade Federal do Estado do Rio de Janeiro (UNIRIO), Av. Pasteur, 458 – Sala 314A, CEP 22290-240, Rio de Janeiro, RJ, Brazil.

^2^ Programa de Pós-Graduação em Ecologia e Evolução (PPGEE), Universidade do Estado do Rio de Janeiro (UERJ), Rua São Francisco Xavier, 524, CEP 20550- 900, Rio de Janeiro, RJ, Brazil.

^3^ Programa de Pós-graduação em Biodiversidade Neotropical (PPGBIO), Universidade Federal do Estado do Rio de Janeiro (UNIRIO), Av. Pasteur, 458 – Sala 506A, CEP 22290-240, Rio de Janeiro, RJ, Brazil.

Corresponding Author:

Vanessa Bettcher^1^

Programa de Pós-Graduação em Ecologia e Evolução (PPGEE), Universidade do Estado do Rio de Janeiro (UERJ), Rua São Francisco Xavier, 524, CEP 20550- 900, Rio de Janeiro, RJ, Brazil.

Email address: vanessa_bettcher@hotmail.com

#

# Appendix 2 - List of the studies obtained through a literature review of the habitat association of the Atlantic Nurse Shark *Ginglymostoma cirratum* using two groups of keywords: (1) “habitat” AND ("Ginglymostoma cirratum" OR "Squalus punctulatus" OR "Squalus cirrhatus" OR "Ginglymostoma cirrhatum" OR "Squalus punctatus" OR "Scyllium cirrhosum" OR "Ginglymostoma cirrosum" OR "Squalus argus" OR "Ginglymostoma fulvum" OR "Ginglymostoma caboverdianus" OR "Ginglymostoma cirrotum" OR "nurse shark"); and (2) (“Abiotic” OR "Habitat") AND ("Ginglymostoma cirratum" OR "Squalus punctulatus" OR "Squalus cirrhatus" OR "Ginglymostoma cirrhatum" OR "Squalus punctatus" OR "Scyllium cirrhosum" OR "Ginglymostoma cirrosum" OR "Squalus argus" OR "Ginglymostoma fulvum" OR "Ginglymostoma caboverdianus" OR "Ginglymostoma cirrotum") at the Dimensions research database.

Adams, D.H. and R. Paperno. (2007). Preliminary assessment of a nearshore nursery ground for the scalloped hammerhead off the Atlantic Coast of Florida. American Fishery Society Symposium 50: 165-174

Afonso, A. S., Andrade, H. A., & Hazin, F. H. V. (2014). Structure and Dynamics of the Shark Assemblage off Recife, Northeastern Brazil. PLoS ONE, 9(7), e102369. doi:10.1371/journal.pone.0102369

Afonso, A. S., Cantareli, C. V., Levy, R. P., Veras, L. B. (2016). Evasive mating behaviour by female nurse sharks, *Ginglymostoma cirratum* (Bonnaterre, 1788), in an equatorial insular breeding ground. Neotropical Ichthyology, 14(4). doi:10.1590/1982-0224-20160103

Bruns, S., and Henderson, A. C. (2020). A baited remote underwater video system (BRUVS) assessment of elasmobranch diversity and abundance on the eastern Caicos Bank (Turks and Caicos Islands); an environment in transition. Environmental Biology of Fishes. doi:10.1007/s10641-020-01004-4

Carrier, J.C., and Pratt, H.L. (1998). Habitat Management and Closure of a Nurse Shark Breeding and Nursery Ground. Fisheries Research 39(2):209–13. doi: 10.1016/s0165-7836(98)00184-2.

Castro J.I. (2000). The biology of the nurse shark, *Ginglymostoma cirratum*, off the Florida east coast and the Bahama Islands. Environmental Biology of Fishes 58: 1–22

Chapman, D.D. & Pikitch, E.K. & Babcock, F., Shivji, M.S. (2005). Marine Reserve Design and Evaluation Using Automated Acoustic Telemetry: A Case-Study Involving Coral Reef-Associated Sharks in the Mesoamerican Caribbean. Marine Technology Society Journal 39(1):42–55. doi: 10.4031/002533205787521640.

DeAngelis B., McCandless C., Kohler N., Recksiek C., Skomal G.B. (2008). First characterization of shark nursery habitat in the United States Virgin Islands: evidence of habitat partitioning by two shark species. Mar Ecol Prog Ser 358:257–271. https://doi.org/10.3354/meps07308

Eggleston, David B., and Romuald N. Lipcius. (1992). Shelter Selection by Spiny Lobster Under Variable Predation Risk, Social Conditions, and Shelter Size. Ecology 73(3):992–1011. doi: 10.2307/1940175.

Ferreira, Luciana C., André S. Afonso, Pedro C. Castilho, Fábio H. V Hazin. (2013). Habitat Use of the Nurse Shark, *Ginglymostoma cirratum*, off Recife, Northeast Brazil: A Combined Survey with Longline and Acoustic Telemetry. Environmental Biology of Fishes 96(6):735–45. doi: 10.1007/s10641-012-0067-5.

Garla, R. C., Garcia, J., Veras, L. B., Lopes, N. P. (2009). Fernando de Noronha as an insular nursery area for lemon sharks, *Negaprion brevirostris*, and nurse sharks, *Ginglymostoma cirratum*, in the equatorial western Atlantic Ocean. Marine Biodiversity Records, 2. doi:10.1017/s1755267209000670

Garla, R. C., Garrone-Neto D., Gadig, O.B.F. (2014). Defensive Strategies of Neonate Nurse Sharks, *Ginglymostoma cirratum*, in an Oceanic Archipelago of the Western Central Atlantic. Acta Ethologica 18(2):167–71. doi: 10.1007/s10211-014-0200-x.

Gilmore, R. G. Jr, Donohoe, C. J., Cooke, D. W. & Herrema, D. J. (1981). Fishes of the Indian River Lagoon and adjacent waters. Harbor Branch Foundation Technical Report 41, 64 pp.

Gutowsky, L. F. G., M. James Rider, R. P. Roemer, A. J. Gallagher, M. R. Heithaus, S. J. Cooke, and N. Hammerschlag. (2021). Large Sharks Exhibit Varying Behavioral Responses to Major Hurricanes. Estuarine Coastal and Shelf Science 256:107373. doi: 10.1016/j.ecss.2021.107373.

Hannan, K. M., Driggers III, W. B., Hanisko, D. S., Jones, L. M., & Canning, A. B. (2012). Distribution of the Nurse Shark, *Ginglymostoma cirratum*, in the Northern Gulf of Mexico. Bulletin of Marine Science, 88(1), 73–80. doi:10.5343/bms.2011.1033

Hansell, A. C., Kessel, S. T., Brewster, L. R., Cadrin, S. X., Gruber, S. H., Skomal, G. B., & Guttridge, T. L. (2018). Local indicators of abundance and demographics for the coastal shark assemblage of Bimini, Bahamas. Fisheries Research, 197, 34–44. doi:10.1016/j.fishres.2017.09.016

Lennon, E., and Sealey, K. S. (2021). Elasmobranch (Chondrichthyes, Elasmobranchii) Habitat Use in an Insular Tropical Lagoon in Exuma, The Bahamas. Caribbean Journal of Science 51(1): 20-29. https://doi.org/10.18475/cjos.v51i1.a3

Niella, Y.V, Hazin, F.H.V., Afonso, A.S. (2017). Detecting Multispecific Patterns in the Catch Composition of a Fisheries‐Independent Longline Survey. Marine and Coastal Fisheries 9(1):388–95. doi: 10.1080/19425120.2017.1347115.

Pikitch E.K., Chapman D.D., Babcock E.A., Shivji MS. (2005). Habitat use and demographic population structure of elasmobranchs at a Caribbean atoll (Glover’s Reef, Belize). Mar Ecol Prog Ser 302: 187−197

Pratt H.L., and Carrier, J.C. (2007). The nurse shark, mating and nursery habitat in the Dry Tortugas, Florida. In American Fisheries Society Symposium (Vol. 50, p. 225). American Fisheries Society

Pratt, H. L., Pratt, T. C., Morley, D., Lowerre-Barbieri, S., Collins, A., Carrier, J. C., et al. Whitney, N. M. (2018). Partial migration of the nurse shark, *Ginglymostoma cirratum* (Bonnaterre), from the Dry Tortugas Islands. Environmental Biology of Fishes, 101(4), 515–530. doi:10.1007/s10641-017-0711-1

Shipley, O. N., Murchie, K. J., Frisk, M. G., O’Shea, O. R., Winchester, M. M., Brooks, E. J., et al. Power, M. (2018). Trophic niche dynamics of three nearshore benthic predators in The Bahamas. Hydrobiologia, 813(1), 177–188. doi:10.1007/s10750-018-3523-1

Smith, K. N., and Herrkind, W. F. (1992). Predation on early juvenile spiny lobsters *Panulirus argus* (Latreille): influence of size and shelter. Journal of Experimental Marine Biology and Ecology, 157(1), 3–18. doi:10.1016/0022-0981(92)90070-q

Stoffers, T., de Graaf, M., Winter, H. V, & Nagelkerke, L. A. J. (2021). Distribution and ontogenetic habitat shifts of reef associated shark species in the northeastern Caribbean. Marine Ecology Progress Series, 665, 145–158. https://doi.org/10.3354/meps13688

Rangel, B. S., Hammerschlag, N., Moreira, R. G. (2021). Urban Living Influences the Nutritional Quality of a Juvenile Shark Species. The Science of The Total Environment 776:146025. doi: 10.1016/j.scitotenv.2021.146025.

Smith, K.N., and Herrkind, F.W. 1992. Predation on Early Juvenile Spiny Lobsters *Panulirus Argus* (Latreille): Influence of Size and Shelter. Journal of Experimental Marine Biology and Ecology 157(1):3–18. doi: 10.1016/0022-0981(92)90070-q.

Springer, S. (1950). An outline for a Trinidad shark fishery. Proceedings of the Gulf and Caribbean Fisheries Institute. Second Annual Session. Univ. of Miami, Coral Gables. pp. 17-26.

Tinari, A.M., and Hammerschlag, N. 2021. An Ecological Assessment of Large Coastal Shark Communities in South Florida. Ocean & Coastal Management 211:105772. doi: 10.1016/j.ocecoaman.2021.105772.

Ward-Paige, C.A., Mora, C., Lotze H.K., Pattengill-Semmens, C., McClenachan, L., Arias-Castro, E., and Myers, R.A. 2010. Large-Scale Absence of Sharks on Reefs in the Greater-Caribbean: A Footprint of Human Pressures. PLOS ONE 5(8):e11968. doi: 10.1371/journal.pone.0011968.

Wiley T.R., and Simpfendorfer C.A. (2007). The ecology of elasmobranchs occurring in the Everglades National Park, Florida: implications for conservation and management. Bull Mar Sci 80: 171−189
